# Surgical Management of Pleomorphic Lung Carcinoma With Left Atrial Invasion: Two Cases Including One With Cerebral Artery Metastasis

**DOI:** 10.1111/1759-7714.70199

**Published:** 2025-12-26

**Authors:** Eitetsu Koh, Yasuo Sekine, Hiroyuki Saitou, Kenzo Hiroshima

**Affiliations:** ^1^ Department of Thoracic Surgery Tokyo Women's Medical University Yachiyo Medical Center Chiba Japan; ^2^ Department of Cardiovascular Surgery Tokyo Women's Medical University Yachiyo Medical Center Chiba Japan; ^3^ Department of Pathology Tokyo Women's Medical University Yachiyo Medical Center Chiba Japan

**Keywords:** cardiopulmonary bypass, cerebral aneurysm, left atrial invasion, lung cancer surgery, pleomorphic carcinoma

## Abstract

Pleomorphic carcinoma is a rare, aggressive subtype of non‐small cell lung cancer (NSCLC). Invasion into the left atrium and dissemination to cerebral arteries are exceptionally uncommon, and the role of cardiopulmonary bypass (CPB)–assisted resection remains debated. We report two surgically treated cases with left atrial invasion. Case 1: A 57‐year‐old man underwent left lower lobectomy with partial atrial resection under CPB. One month later, he developed subarachnoid hemorrhage caused by rupture of a cerebral aneurysm secondary to metastasis; histology of the aneurysmal wall confirmed carcinoma. He remains recurrence‐free at 21 months. Case 2: A 62‐year‐old woman underwent extended left upper lobectomy with partial atrial resection under CPB. Although adrenal metastasis was suspected radiologically, pathological confirmation was lacking preoperatively; surgery was pursued because of symptomatic disease and atrial involvement. She developed postoperative cerebral infarction and rapid adrenal progression and died at 4 months despite chemotherapy. These cases illustrate both the technical feasibility of CPB‐assisted atrial resection and the aggressive biology of pleomorphic carcinoma, including atypical vascular metastasis to cerebral arteries. Careful staging, patient selection, and early multidisciplinary planning (thoracic surgery, cardiac surgery, neurosurgery, oncology, and radiology) are essential. Surgery can be justified in selected patients with atrial invasion; however, pleomorphic histology portends poor outcomes and unusual metastatic tropism. Vigilant postoperative surveillance and integration of systemic therapy are required.

## Graphical abstract

Two cases of pleomorphic lung carcinoma (PLC) with left atrial invasion are presented, including one complicated by a ruptured metastatic cerebral aneurysm causing subarachnoid hemorrhage. Both patients underwent one‐stage en bloc pulmonary and atrial resection under cardiopulmonary bypass (CPB). These cases illustrate that CPB can enable complete resection for selected PLCs with intracardiac extension. The occurrence of a metastatic cerebral aneurysm highlights the tumor's vascular tropism/EMT‐like behavior and the need for vigilant neurovascular assessment and multidisciplinary perioperative planning.

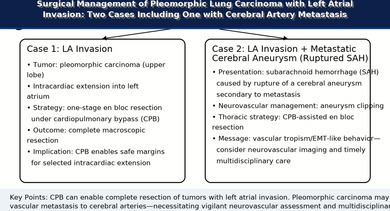

## 1 Introduction

Pleomorphic carcinoma accounts for < 1% of NSCLC and is characterized by spindle and/or giant cells with high metastatic potential and chemoresistance. When tumors extend into the left atrium, CPB‐assisted resection is occasionally attempted, but long‐term oncologic benefit is uncertain. Metastatic involvement of cerebral arterial walls leading to aneurysmal rupture is exceedingly rare. We present two pleomorphic carcinoma cases with atrial invasion—one complicated by histology‐proven metastatic cerebral aneurysm—to discuss surgical feasibility, selection, and surveillance.

## 2 Case Presentations

### 2.1 Case 1

**FIGURE 1:**
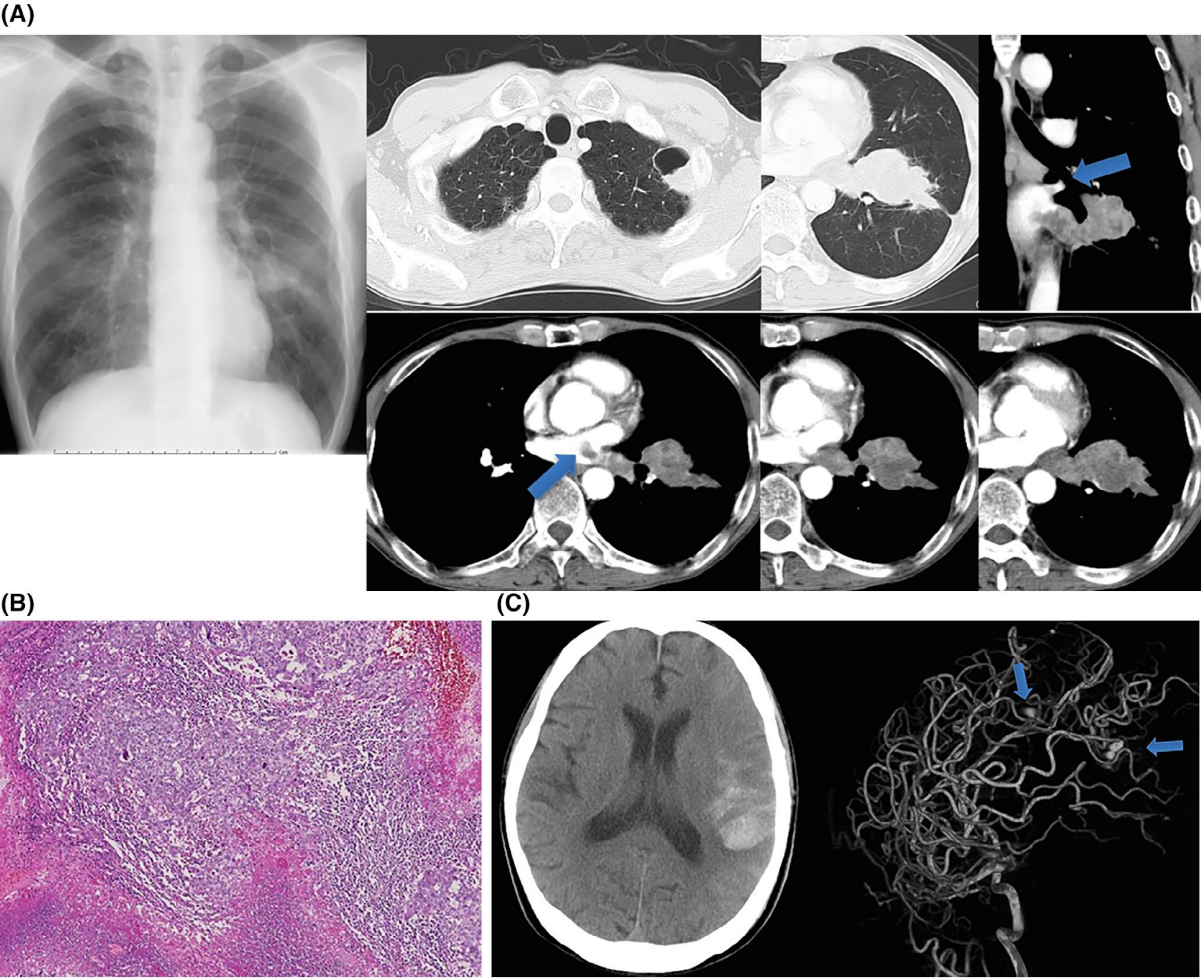
Case 1. (A) Preoperative chest radiograph/CT showing a left lower‐lobe mass invading the left atrium (arrows); EBUS‐TBNA supported surgery by excluding mediastinal nodal disease. Clinical stage cT4N0/1 M0, stage IIIA. (B) Histology (H&E): Pleomorphic carcinoma with marked atypia and spindle cells; pT4N0M0, pl0, pm2. (C) Postoperative CT/MRA revealing multiple cerebral aneurysms (arrows); enlargement despite antibiotics prompted clipping. Aneurysmal wall contained metastatic carcinoma.

A 57‐year‐old man presented with hemoptysis and frequent atrial arrhythmias. CT showed a left lower‐lobe mass invading the left atrium. Endobronchial ultrasound–guided nodal assessment showed no mediastinal metastasis. Due to urgent symptoms (recurrent hemoptysis/arrhythmias), surgery proceeded without cranial MRI. Left lower lobectomy with partial atrial resection under CPB was performed. Pathology: pleomorphic carcinoma (pT4N0M0, pl0, pm2, and stage IIIA). One month later, subarachnoid hemorrhage occurred; angiography revealed multiple aneurysms. Aneurysm clipping confirmed metastatic carcinoma in the aneurysmal wall. At 21 months postoperatively, he remains recurrence‐free (Figure 1A–C).

### 2.2 Case 2

**FIGURE 2:**
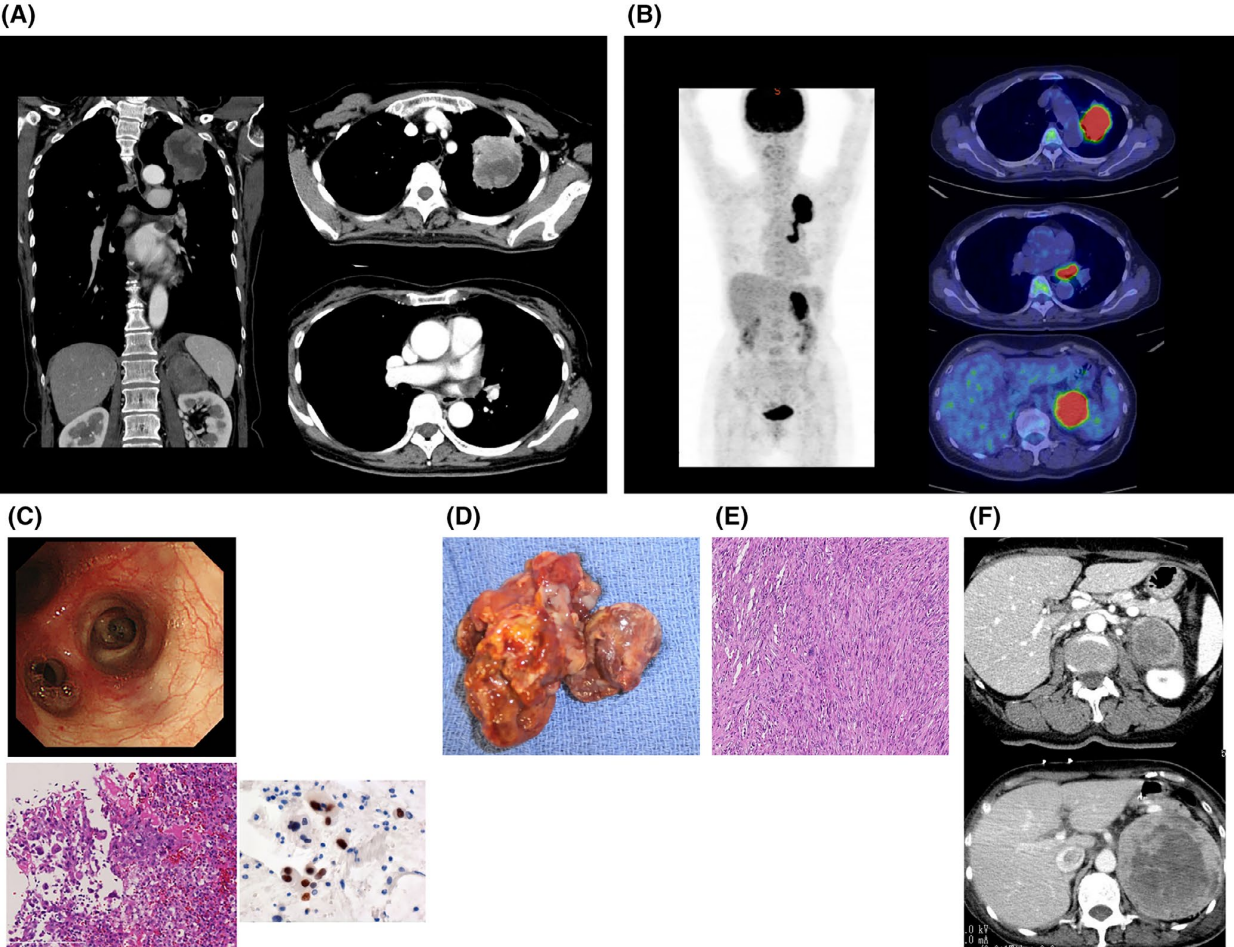
Case 2. (A) Preoperative CT: 6‐cm left upper‐lobe mass with pulmonary vein/atrial invasion. (B) PET‐CT: High uptake in the lung tumor and left adrenal gland (SUVmax 24.5 → 27.5). (C) Bronchoscopy/biopsy: Poorly differentiated NSCLC with pleomorphism; IHC TTF‐1+, p40−, Napsin A−, p63−; EGFR mutation negative. (D) Intraoperative gross: ~4.5‐cm mass protruding into the atrial cavity. (E) Histology (H&E): Pleomorphic carcinoma; pT4N0. (F) Abdominal CT: Adrenal lesion grew from ~4 cm preop to > 10 cm at 3 months.

A 62‐year‐old woman had a 6‐cm left upper‐lobe mass extending into the left superior pulmonary vein and left atrium. PET‐CT suggested adrenal uptake; preoperative pathology confirmed pleomorphic carcinoma while adrenal metastasis was unproven histologically. Extended left upper lobectomy with partial atrial resection under CPB was performed. She developed postoperative cerebral infarction and rapid adrenal progression despite carboplatin–paclitaxel chemotherapy, and died 4 months postoperatively (Figure 2A–F).

## 3 Discussion

Our experience underscores dual challenges: the technical demands of atrial resection and the intrinsically aggressive biology of pleomorphic carcinoma. Small series have reported acceptable perioperative outcomes with atrial resection under CPB in selected T4 NSCLC, but durable control is limited in histologies with early dissemination. In Case 1, metastatic infiltration of a cerebral arterial wall caused aneurysmal rupture—an extremely rare pattern previously described only sporadically. From a biological perspective, pleomorphic carcinoma frequently shows epithelial–mesenchymal transition features, aligning with early hematogenous spread and atypical vascular tropism. Clinically, we recommend comprehensive preoperative staging (including brain imaging when feasible), explicit documentation of nodal assessment, early involvement of cardiac surgery, and vigilant neuro‐oncologic surveillance. In suspected stage IV disease, the threshold for induction or systemic therapy should be low; surgery must rest on multidisciplinary consensus and patient‐centered goals [1, 2, 3, 4].

## 4 Conclusions

CPB‐assisted resection can be feasible for left‐atrial invasion; however, pleomorphic histology often dictates outcome. Multimodal strategies and close surveillance are essential, and unusual vascular metastases should be anticipated.

## Author Contributions

Conceptualization: Eitetsu Koh, Yasuo Sekine. Methodology: Eitetsu Koh, Hiroyuki Saito. Surgery/clinical management: Eitetsu Koh, Yasuo Sekine, Hiroyuki Saito. Pathology Investigation: Kenzo Hiroshima. Data curation: Eitetsu Koh. Writing – original draft: Eitetsu Koh. Writing – review and editing: All authors. Supervision: Yasuo Sekine.

## Funding

The authors have nothing to report.

## Ethics Statement

This retrospective case report complied with institutional and national ethical standards. Institutional review board approval was waived.

## Consent

Written informed consent for participation and publication (clinical details and images) was obtained from both patients.

## Conflicts of Interest

The authors declare no conflicts of interest.

## Data Availability

All data supporting this report are included in the article and its Supporting Information.
